# The Relationship between Obesity and Pre-Eclampsia: Incidental Risks and Identification of Potential Biomarkers for Pre-Eclampsia

**DOI:** 10.3390/cells11091548

**Published:** 2022-05-05

**Authors:** Talitha Abraham, Andrea M. P. Romani

**Affiliations:** Department of Physiology and Biophysics, School of Medicine, Case Western Reserve University, 10900 Euclid Avenue, Cleveland, OH 44106-4970, USA; talitha.abraham@case.edu

**Keywords:** pre-eclampsia, obesity, biomarkers, adipokines, adiponectin, leptin, ROS, angiogenic factors

## Abstract

Obesity has been steadily increasing over the past decade in the US and worldwide. Since 1975, the prevalence of obesity has increased by 2% per decade, unabated despite new and more stringent guidelines set by WHO, CDC, and other public health organizations. Likewise, maternal obesity has also increased worldwide over the past several years. In the United States, pre-pregnancy rates have increased proportionally across all racial groups. Obesity during pregnancy has been directly linked to obstetric complications including gestational diabetes, HTN, hematomas, pre-eclampsia, and congenital defects. In the particular case of pre-eclampsia, the incidence rate across the globe is 2.16%, but the condition accounts for 30% of maternal deaths, and a robust body of evidence underscored the relationship between obesity and pre-eclampsia. More recently, attention has focused on the identification of reliable biomarkers predictive of an elevated risk for pre-eclampsia. The aim of this literature review is to elucidate the relationship between obesity and these predictive biomarkers for future prediction and prevention of pre-eclampsia condition in women at risk.

## 1. Introduction

Obesity has been steadily increasing over the past decade in the U.S. and worldwide [1]. Starting in 1975, the prevalence of obesity has increased by 2% per decade [1] but has remained largely dismissed or ignored until a decade ago, when progressively more attention has been paid to this public health condition owing to the large number of individuals affected and the associated complications [1,2]. First reported as an epidemic in the U.S., obesity quickly earned the label of pandemic due to its rapid progression and spreading to other developed and developing nations [3]. A rapid increase in upward trend was first noted as early as 1988, and since then prevalence has increased from 22.9% to 30.5% by the year 2000 [1,2]. Concomitantly, morbid obesity has also increased by 2%. Recent reports from the WHO and the CDC indicate that approximately 1 billion people worldwide present with obesity at various stages, regardless of age, gender, and ethnicity [4,5,6]. According to the National Institutes of Health (NIH), obesity is the second leading cause of preventable death in the U.S. causing 300,000 deaths annually [7].

Maternal obesity has also increased significantly over the past decade, in line with the general uptrend of obesity. In 1980, 29.8% of women were overweight or obese, and by 2013 the incidence rate had increased to 38% in both high- and middle-income countries [8]. In the U.S., pre-pregnancy rates have increased by 11% between 2016 and 2019, and this increase has occurred proportionally across all racial groups [2]. Obesity during pregnancy has been linked primarily to obstetric complications including gestational diabetes, HTN, hematomas, pre-eclampsia, and congenital defects [9]. Further, a strong association has been observed between increasing BMI and risk of still-birth, with obesity being responsible for 25% of stillbirths between 37- and 42-week gestation [10].

A significant body of studies has investigated the systemic effects of obesity, its role in multiple comorbidities, and the effects of high BMI on mortality [11]. Research has also highlighted the relationship between obesity and pre-eclampsia [12,13]. At the same time, a large body of research has attempted to identify possible biomarkers predictive of an elevated risk for pre-eclampsia. Incidence of several adverse pregnancy outcomes are associated with increased weight gain. This is particularly true for pre-eclampsia. Pre-eclampsia is observed in 2–8% of pregnancies globally. Multiple studies have confirmed that maternal obesity increases the risk of pre-eclampsia by three-four times when compared to normo-weight mothers [14,15,16]. This increased risk is especially concerning if we consider that at front of an average incidence rate of 2.16% across the globe as an obstetric complication, pre-eclampsia accounts for 30% of maternal deaths [17]. By monitoring more than 39 million births over 10 years, the National Hospital Discharge Survey has estimated to ~6% the incidence of hypertensive disorders in pregnancy, which include pre-eclampsia, eclampsia, gestational hypertension and chronic hypertension, and between 3- and 25-fold the increased risk of severe pregnancy complications in women with pre-eclampsia and eclampsia [18].

The goal of this literature review is to elucidate the current notion in the field that obesity is a major risk factor for pre-eclampsia, and discuss the potential biomarkers that can predict pre-eclampsia onset and progression in over-weight mothers as compared to normo-weight ones. In particular, this review aims at determining whether abnormalities in the levels of the currently most accepted biomarkers for pre-eclampsia are related, directly or indirectly, to obesity and/or the underlying dysmetabolic conditions associated with weight gain, overweight, or frank obesity in pregnant women.

**Figure 1:**
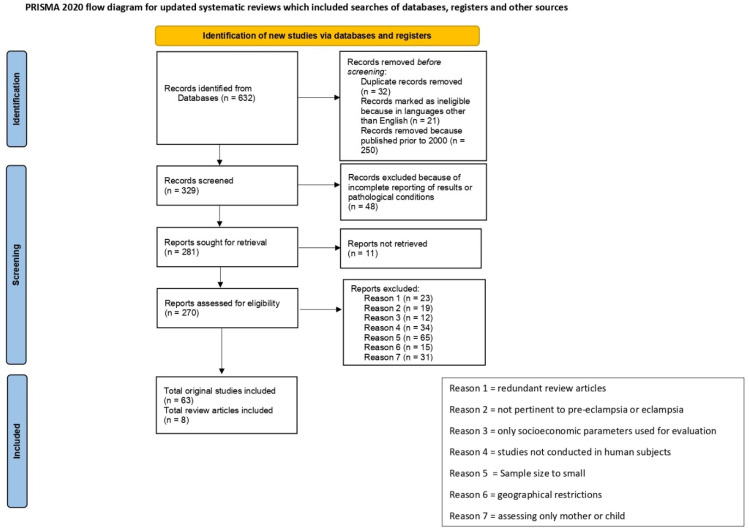
The flow chart illustrates the number of articles identified through the literature search and the inclusion/exclusion screening criteria to select the pertinent articles to be included in the review.

For this review, recent literature was collected using online databases (MEDLINE and BIOMED) searches. Used keywords included: gestational weight gain, sugary dietary consumption, pre-eclampsia risk, and biomarkers, such as adiponectin, leptin, resistin, vascular endothelial growth factor (VEGF), placental growth factor (PlGF), soluble fms-like tyrosine kinase (sFlt1), soluble endoglin (sEng), oxidative stress, and HLA-antigens. A total of 632 articles were initially obtained. Articles were excluded based on the following: publication date prior to 2000 (to be more in tune with the current biomedical perception of the disease); studies addressing only obesity or pre-eclampsia but not both conditions; assessment of only long term implications for mother and child; studies with limited or too small subject groups, or with subject groups selected based on criteria that strictly applied to particular geographical areas (e.g., exposure to local diseases, limited access to health care, reliance on traditional medicine, cultural beliefs); studies using exclusively age or socioeconomic status of the mothers without mention or consideration of biological risk-factors; studies conducted on non-human subjects only; absence or incomplete reporting of results. These exclusion criteria (Figure 1) reduced the number of utilizable articles to 63 original articles plus 8 review articles. Of the 63 articles, 8 addressed adipokines, 6 use of sugary dietary products, 14 serum factors, 2 histocompatibility antigens, 23 obesity and 10 pre-eclampsia or eclampsia. The references listed include populations from Northern Sweden, USA, Tanzania, China, Northwest Ethiopia, Saudi Arabia, Northeast Brazil, Norway, Denmark, and Australia, with sample size varying from 60 to 33,000. The major findings of each study reported in Table 1 will be discussed in this review.

## 2. Pathophysiology of Obesity and Pre-Eclampsia and Discussion of the Findings

To better frame the findings reported in Table 1, the main pathophysiological and clinical observations relative to obesity and pre-eclampsia will be briefly summarized here, before discussing the main findings reported in the cited literature.

## 3. Obesity

Obesity is defined as a medical condition in which excess body fat has accumulated in an individual to an extent that it may have a negative effect on the individual’s health. Metabolic imbalance as observed in the metabolically active fat surrounding the abdominal organs, i.e., visceral fat, is implicated in metabolic dysregulation [47], and the greater is the amount of adiposity accumulated, the higher and more complex are the associated metabolic issues [47]. The main criterion used to determine obesity in an individual is the BMI or body mass index (kg/height in m^2^). According to NIH guidelines [https://www.nhlbi.nih.gov/health/educational/lose_wt/BMI/bmi_tbl.pdf (accessed on 10 April 2022)], an individual is defined as normo-weight when the BMI is between 19 and 24.9, overweight when the BMI is between 25 and 29.9, obese when the BMI is between 30 and 39.9, and morbidly obese when the BMI is 40 or higher. Additionally, central obesity is used as a criterion to assess cardiovascular risk. Abdominal circumference above 94 cm in men and 80 cm in women indicate increased cardiovascular risk [19,48], although this concept has recently been revised to take into account race and ethnicity [47]. Several risk factors are considered in the propensity to develop obesity, including genetics and dietary regimen, but primarily lifestyle habits. The most common genetic markers are responsible for <1.5% cases of obesity. High caloric diet, reduced physical activity, extreme amounts of sleep, and socio-economic status play a larger role as risk factors [47].

Metabolic syndrome is a particular health condition associated with obesity. This syndrome is characterized by a set of pathologies that co-exist in a certain patient. These pathologies include central obesity, insulin resistance with higher than normal fasting glycemia, HTN, liver steatosis, and dislipidemia, which together with chronic inflammation can lead to aggressive atherosclerosis [49]. Diabetes and insulin resistance are often present and are considered to be the result of lipotoxicity [50]. Triacylglycerols deposition within adipocytes provide a protective function in low BMI individuals by preventing the circulation of free fatty acids and their biochemical conversion by oxidative stress. At higher BMIs, where there is excess energy storage, the sympathetic nervous system is activated [51], leading to lipolysis and increased free fatty acid release into circulation. Accumulation of poorly utilized lipids within tissues like liver, heart, and skeletal muscles, among others, promotes lipotoxicity and tissue damage. Insulin-receptor in these tissues is among the most common sites of damage, and the ensuing dysfunction of the receptor-together with β-islet cells fatigue as an attempt to increase insulin output to counteract elevated blood glucose levels [50]-result in an insulin-resistant state that further exacerbates the existing hyperglycemia.

On the other hand, HTN and chronic inflammation are the result of the release of cytokines from adipocytes [50]. The adipokines released include interleukin-1, interleukin-6, resistin and TNF-α (tumor necrotic factor-alpha), the latter two being directly involved in promoting insulin resistance. Other adipokines involved are leptin, whose release is increased, and adiponectin, the release of which is reduced [52]. As adiponectin antagonizes Angiotensin-II, its decrease can explain, at least in part, the hypertension observed in the metabolic syndrome but also in pre-eclampsia in obese mothers [50].

The increased circulation of inflammatory adipokines, in particular interleukin-1 and interleukin-6, give rise to multiple pathologies that share an inflammatory base. Among these pathologies, polycystic ovary syndrome, depression, infertility, and pre-eclampsia are the main clinical conditions associated with pregnancy [50].

## 4. Pre-Eclampsia

Pre-eclampsia is an obstetric complication affecting 2–8% of pregnancies globally [11]. This multisystem progressive disorder is characterized by “…the new onset of hypertension and proteinuria, or the new onset of hypertension and significant end-organ dysfunction with or without proteinuria in the last half of pregnancy or postpartum…” (https://www.uptodate.com/contents/preeclampsia-clinical-features-and-diagnosis; accessed 10 April 2022). Hypertension and proteinuria resolve following child delivery or by the 6th week postpartum [53]. Pre-eclampsia and its immediate complications, including eclampsia, are responsible for 10 to 15% of maternal deaths worldwide [54]. Because of this high death toll, the pathology continues to be extensively investigated through the lens of obesity being an important, modifiable risk factor. In fact, according to a prospective cohort study carried out in 2005, pre-eclampsia risk increases at least three folds in women with a BMI of 30 as compared to women with normal (i.e., <24.9) BMI [14].

While the etiology of pre-eclampsia is still not fully elucidated, abnormal placentation due to defective invasion of cytotrophoblast by the spiral arteries has been pinpointed to play a causal role [53]. It has been suggested that inhibition of nitric oxide synthesis is involved in the abnormal placentation through increased arterial resistance [55]. The resulting oxidative stress induces a release of cytokines, oxidized lipids, and free radicals that directly affect the functionality of the vascular endothelium [56]. The ensuing endothelial dysfunction and the abnormalities in Angiotensin-II regulation mentioned previously [50] have been invoked to cause the elevated blood pressure observed in pre-eclampsia. The effects of elevated systolic pressure on endothelial cells extends to the glomerular filtration barrier in the kidneys. The associated depletion of vascular endothelial growth factors in the podocytes further compromises the glomerular filtration process, giving rise to proteinuria [53]. The progressive imbalance between increased systolic pressure and decreased oncotic pressure, and the hyper-permeability of the vascular endothelium can then explain the insurgence of edemas in the lower extremities and the lungs, typical symptoms and complications of pre-eclampsia.

The maternal immune system is also highly involved in pre-eclampsia pathophysiology. Due to lack of recognition of the feto-placental unit, immune cells are overproduced, leading to elevated TNF-α levels and inducing apoptosis of the cytotrophoblast [53,57]. The histocompatibility antigen HLA-G is important for correct invasion of the cytotrophoblast, and its expression is reduced in all forms of pre-eclampsia [58]. The interactions between HLA-G and cytotrophoblasts are possibly mediated by VEGF and placental growth factors, and it has been proposed that the levels of all these growth factors are strong predictors of pre-eclampsia [53]. Because these biological, vascular, and immunological components appear to contribute differently to the etiology of pre-eclampsia, recent literature divides pre-eclampsia in different types/subtypes, each characterized by distinct pathophysiological processes, risk factors, clinical outcomes, and long-term prognosis [10]. One of the most commonly proposed distinctions is between early-onset (before the 34th week of gestation) versus late-onset (34th week of gestation, or later) based on the spiral artery remodeling, which is present in the early-onset but not in the late-onset [10].

Aside for risk of pre-eclampsia progressing to eclampsia in the short-term, the long-term effects of pre-eclampsia on both mother and child are also significant. The mortality risk for mothers who experience pre-eclampsia is elevated primarily as a result of cardiovascular complications [20]. Some of these complications can persist for a very long time, as pre-eclampsia has been reported to cause cardiovascular complications and cardiac diseases in the mother 20 years after the pre-eclampsia event. As for the children delivered by pre-eclamptic mothers, they are usually small in size and with low birth weight, conditions that can lead to increased risk for coronary artery disease and other cardiovascular etiologies later in life [53,59].

## 5. The Effect of Obesity on Risk for Pre-Eclampsia

By now, it is a well-accepted notion that an elevated BMI as observed in over-weight and obese women represents a major risk factor for pre-eclampsia (see ref. [9] as an example). It is less clear, however, to which extent obesity promotes the onset of other biological parameters identified as potentially clinically relevant risk-factors to predict pre-eclampsia and its impact on maternal and newborn health.

Our analysis of the literature cited here reaffirms the notion that obesity imposes major significant negative effects on pregnancy, directly, and indirectly through the associated metabolic dysfunctions and the increase in basal inflammatory state. Both these conditions can lead to various obstetric complications such as gestational diabetes and hypertension, thus setting a baseline of increased pre-eclampsia risk.

Consistent with this assertion, the majority of the studies reported in Table 1 substantiate that overweight and obese women have a higher risk for pre-eclampsia as compare to normo-weight mothers. A retrospective study carried out in 2014 at King Khalid University Hospital grouped mothers by BMI levels and presence of gestational diabetes (GDM) to investigate the independent effects of GDM and obesity on adverse pregnancy outcomes. The results obtained from about 2700 women assessed clearly showed the independent negative effects of obesity and GDM on pregnancy outcomes including pre-eclampsia. Interestingly, the risk for adverse outcomes increased synergistically when both obesity and GDM were present as compared to when only one of these two conditions was present. In agreement with the trend observed in many Arab countries in the last decade, the study reported an incidence of maternal obesity of 44%, which the investigators attributed to the increased sedentary lifestyle in 75% of the participants [21].

Similar results were reported by the registry-based 2020 study carried out in Tanzania in more than 17,000 women and in a similar study carried out in Sweden. The Tanzanian study confirmed the strong association between pre-eclampsia and obesity as overweight and obese women presented higher risk of pre-eclampsia [22]. This association was independent of the socio-economic status of the women as it was observed in both wealthy and low-income communities [22]. The Swedish birth-registry study, which actually preceded the Tanzanian study, corroborated the importance of obesity as a risk factor in that elevated BMI increased the risk for all types of pre-eclampsia and its complications including delivery at term [23]. Similar support is provided by the prospective study performed in Brazil in 2013 [24]. Out of the initial 212 women recruited for the study, 30 suffered pre-eclampsia (approximately 14%). On average, these women had higher BMI values than the normo-weight, normotensive controls. By including a socioeconomic stratification, this study evidenced that women who developed pre-eclampsia had lower levels of education than normotensive women, as only 20% of them had completed high school. This discrepancy in outcome was attributed to reduced access to quality health care and poor compliance to treatment and nutrition [24].

Aside from pre-pregnancy obesity, weight gain during pregnancy (gestational weight gain or GWG) has been implicated in adverse pregnancy outcomes. Weight gain during pregnancy should occur within the normal range based on the pre-pregnancy BMI of the mother and the expected development and growth of the placenta and the fetus. Gestation weight gain in excess of the predicted range appears to lead to detrimental pregnancy outcomes. The results of a recent retrospective cohort study involving 1606 women with GDM [25] are in agreement with this statement. In this study, the mothers were divided in 2 groups: one group with normal GWG, and a second group with above normal GWG. The results indicated an increased risk of C-section, pre-eclampsia, and pre-term labor in women with above normal GWG and with GDM. One detracting weakness of this study is that while the study focused on GWG, it did not provide weight parameters for the women involved in the study prior to pregnancy, thus preventing from obtaining meaningful insights on the possible link adverse pregnancy outcomes in overweight or obese women [25].

The relevance of weight gain as a risk factor for pre-eclampsia, is also supported by the studies carried out in China and in Northwestern Ethiopia. The cohort study from Lanzou, China [26], supports the notion that gestational weight gain increases the risk of obesity, and pre-pregnancy obesity and gestational weight gain increase the risk for pre-eclampsia, both independently and in conjunction, as the combined effects of maternal obesity and gestational weight gain further heightens the risk for pre-eclampsia. The case-control study from Northwestern Ethiopia [27] included socio-demographic characteristics and diet to the variables tested for pre-eclampsia risk. After controlling for confounding variables such as education, residence, anemia, and alcohol/meat consumption, five variables remained to have a significant impact on pre-eclampsia risk, including coffee consumption, which doubled the risk of pre-eclampsia by elevating systolic blood pressure, but primarily obesity, which showed the strongest association with pre-eclampsia risk especially in younger women (less than 35 years of age) [27].

Congruent with the role of obesity as a risk factor for pre-eclampsia, sedentary lifestyle and sugary food consumption also represents major risk factors of pre-eclampsia in that both contribute to an increase in gestational weight gain. The epidemiology study conducted in North Sweden in 2021 on a sample population of more than 2000 women [28] validates the observation that higher levels of physical activity can reduce the risk of adverse pregnancy outcomes including emergency C-section and gestational weight gain [28]. This notion is supported by the study out of King Khalid University Hospital [21] that identified sedentary lifestyle as the most direct reason behind the high incidence of maternal obesity in the sample population assessed. Consumption of sugary foods and products has also been associated with pre-eclampsia. However, the number of studies addressing this particular aspect is rather limited [29,30,31,32,33]. A prospective study carried out on almost 33,000 normo-weight and overweight pregnant Norwegian women indicated that intake of more than 125 mL of sugary beverages per day was associated with a higher risk of pre-eclampsia as compared to the consumption of sugars as fruit (both dried and fresh) [29]. The results of this study corroborate the observation by Clausen et al. [31] in 3133 pregnant Norwegian women that the risk of pre-eclampsia was increased in women whose sucrose intake covered more than 25% of the total energy intake per day as compared to women whose sucrose intake contributed less than 8.5% to total energy intake. Similarly, a prospective longitudinal cohort study on 55,139 Danish women reported that the incidences of gestational hypertension and pre-eclampsia were strongly associated with a higher adherence to a typical Western diet [34]. At the same time, Schoenaker et al. [30] observed that a Mediterranean-style diet was inversely related to the risk of pregnancy-associated hypertension, including pre-eclampsia, in 3582 Australian women who participated in the 9-year Australian Longitudinal Study on Women’s health.

## 6. Potential Biochemical Biomarkers Linking Obesity to Pre-Eclampsia

Significant attention has been paid to investigating and identifying biomarkers that can consistently predict the development of pre-eclampsia. It is only in the last decade that the clinical practice of measuring multiple markers has started and developed [53,60]. Since 2011, the clinical assessment of different growth factors including placental growth factor, VEGF, and anti-angiogenic markers as endoglin and sFlt1, has become more of a routine in women with pre-eclampsia [53]. It has to be noted, however, that the clinical use of these biomarkers as predictive or diagnostic tools still needs proper validation. Based on our analysis of the literature, it is not surprising that the biomarkers that appear to more reliably predict pre-eclampsia are adipokines associated with obesity and gestational weight gain. A secondary analysis of a randomized control trial in Tanzania [35] assessed leptin levels during pregnancy and its relation to gestational weight gain, and reported that high levels of leptin mid-pregnancy are indeed associated with excessive gestational weight gain [27]. Leptin is a pro-inflammatory adipokine produced mainly by adipocytes and regulated by steroids, which is involved in regulating maternal metabolism but also trophoblast invasion among many other functions [61]. Leptin levels increase during pregnancy due to placenta production of leptin, the levels usually peaking during the second-third trimester [61]. Aside from its effect on gestational weight gain, it is assumed that high levels of leptin play a role in pre-eclampsia’s pathophysiology but the exact mechanisms are not fully elucidated. A case-control study conducted in Hungary associated elevated serum leptin concentrations with increased systolic blood pressure due to its effect on sympathetic activity [36]. A more recent case-control study conducted in 2020 proposed leptin as a strong predictor of pre-eclampsia [37]. However, the absence of a proper assessment of the case numbers and controls based on BMI stratification makes the interpretation of the study rather difficult. Furthermore, several reports in the literature provide contradicting results that do not support the conclusion by Bawah et al. [37]. Elucidating this point of contention and clarifying whether leptin affects the trophoblast vascular invasion directly, or indirectly through abnormalities in maternal metabolism and gestational weight gain remains a wanting topic for future research in the field. A strong linear relationship appears to exist among leptin levels, BMI, and C-reactive protein (CRP) concentrations [36]. Both BMI and CRP levels are elevated with increased leptin concentrations [36]. C-reactive protein is produced by hepatocytes following an increase in the levels of inflammatory cytokines such as interleukin-6, TNF-α, and interleukin-1β, and it is an established inflammatory marker released under various pathological conditions [62]. The inflammatory cytokines that promote CRP release are a direct result of obesity, in that the program switch that occurs under excessive adipose tissue increase results in the release of these cytokines, among others [52].

Under the conditions in which cytokines and leptin levels increase, adiponectin expression and release are reduced [52]. This applies to obesity conditions, and has major implications for the increased systolic blood pressure observed in obese individuals including obese pregnant women. Hence, adiponectin can also be considered a strong predictor of pre-eclampsia risk per se, and even more so when taken together with the changes in leptin and CRP concentrations mentioned above, as a small case-control study completed in 2020 suggests [37]. This study focused on the levels of adiponectin, leptin, resistin, visfatin, and lipids in relation to pre-eclampsia risk and incidence. The study provides further evidence that the combination of reduced adiponectin and increased leptin levels is a strong predictor for pre-eclampsia. Interestingly, decreased levels of adiponectin were the best predictors of pre-eclampsia in those cases in which confounding factors such as age, parity, BMI, and family history of hypertension were controlled. Changes in adiponectin and leptin are known to promote overweight and obesity, and exacerbate insulin resistance in those individuals, lending further support to the notion that obesity and the consequent abnormal whole-body metabolism remains the single most important predictor of the risk of pre-eclampsia. This case-control study is also one of the few studies that investigated the role of resistin and visfatin in inflammation and possibly pre-eclampsia [37]. The relation between these two cytokines and pre-eclampsia, however, is not that clear, and is consistent with previous research reporting unchanged levels of resistin and visfatin in pre-eclampsia [38]. Hence, compared to leptin and adiponectin, resistin and visfatin remain inconsistent and not fully reliable biomarkers for pre-eclampsia prediction at the present time, requiring further and more detailed investigation to attain this role. More detailed investigation is also necessary to determine how rapidly the changes in adiponectin and leptin levels promotes the onset and/or the progression of pre-eclampsia.

While leptin and adiponectin appear to be more reliable predictors of pre-eclampsia in the long-term, the well documented changes in sFlt-1: PIGF ratio are a more dependable biomarker for short-term prediction of pre-eclampsia. The anti-angiogenic sFlt-1 protein is responsible for the endothelial dysfunction observed in pre-eclampsia pathology [63]. PIGF (placenta growth factor) is significantly inhibited by sFlt-1. Hence, the higher the levels of sFlt-1, the lower the levels of PIGF will be, resulting in a high sFlt-1: PIGF ratio [64]. Multiple studies support the notion that a high sFlt-1: PIGF ratio indicates an imminent risk for pre-eclampsia [39,65], and is a very accurate, short-term predictor of pre-eclampsia, especially when the sFlt-1: PIGF ratio is significantly increased [40]. In this regard, the sFlt-1: PIGF ratio has been reported to be a far better predictor for pre-eclampsia than the individual measurements of sFlt-1 and PIGF [41].

Changes in serum lipid profile have also been considered as potential predictors of pre-eclampsia. However, the relation between lipid levels and pre-eclampsia is rather weak [37]. With the exception of a significant reduction in high density lipoprotein (HDL) levels, observed biochemical changes in lipid concentrations and pattern have been attributed to the significant oxidative stress observed in women with pre-eclampsia, which targets lipids but also proteins [37]. The modification of both lipids and proteins by oxidative stress can also explains the endothelial changes observed at the level of the glomerular filtration barrier, which give rise to proteinuria, an objective finding in support of pre-eclampsia diagnosis, and supports the idea of antioxidant therapy as a preventative measure for pre-eclampsia [42]. The effectiveness of such a therapy to reduce the severity of pre-eclampsia and its associated risks, however, remains to be demonstrated.

More recently, inhibin-A [43,44] and oxidative stress [66] have also gain relevance as predictors of pre-eclampsia onset, pre-eclampsia progression as well as fetal growth restriction. The Osredkar’s group has recently reported that when assessed in combination with PIGF or with the sFlt-1/PIGF ratio, inhibin-A markedly and significantly increased the detection rate of pre-eclampsia and pre-eclampsia complicated by fetal growth restriction in most of the cases, the only limitation being cases of fetal growth restriction in deliveries earlier than 34 weeks, wherefore the diagnostic predictive value of inhibin-A was rather limited [43]. Noteworthy, the levels of inhibin-A are increased, and the levels of inhibin-B are decreased in obese women [67].

In accordance with the notion that pre-eclampsia can be caused by abnormal placentation due to the defective invasion of cytotrophoblast by the spiral arteries [53], reduced blood flow and abnormal oxygen utilization have been indicated to result in oxidative stress and dysfunction of placental endothelial nitric oxide synthase, ultimately leading to pre-eclampsia reviewed in [66]. While physiological levels of reactive oxygen species (ROS) are associated with the rapid development of the placenta, supra-physiological levels of ROS together with depletion of antioxidants and abnormalities in superoxide dismutase activity are linked to impaired trophoblast invasion, poor placentation, and pathological waves of hypoxia/reoxygenation. In turn, these conditions promote oxidative stress and production of lipoperoxides that lead to cellular dysfunction, inflammation, and apoptosis [66]. Formation of peroxynitrite (ONOO-) and inhibition of endothelial nitric oxide synthase (eNOS) enzymatic activity are among the most common abnormalities observed in the placenta following an increase in ROS formation [66]. Interestingly, adiponectin has been reported to regulate ROS formation in several tissues [68] including placenta [69].

## 7. Conclusions

The literature review reported here confirms the current view-point that overweight and obese women are at a greater risk for pre-eclampsia than normo-weight women. Our literature review also suggests that the metabolic changes central to obesity’s pathology do pose a significant risk for pre-eclampsia and its adverse outcomes. The reviewed studies consistently support the presence of a direct relationship between BMI and risk of pre-eclampsia, gestational hypertension and gestational diabetes. The relevance of excessive gestational weight gain as a risk factor for pre-eclampsia is further corroborated by the reports indicating that the co-presence of both pre-pregnancy obesity and excessive gestational weight gain results in the highest risk for pre-eclampsia onset and progression. As obesity rates increases steadily in the US and worldwide, the prevalence of pre-eclampsia is likely to continue to increase together with the risk for adverse obstetric outcomes associated with high BMI levels and gestational weight gain. It is therefore crucial that these concerns are communicated clearly and in a timely manner to pre-pregnant and pregnant women, and that risk reduction measure are pursued in all pregnancies involving women with high BMI values.

In addition to obesity and weight gain, a few biomarkers have been identified that heighten the risk for pre-eclampsia onset when present. Among these biomarkers, the strongest short-term predictor of pre-eclampsia is the sFlt-1: PIGF ratio, as several retrospective, and prospective studies clearly indicate. Inhibin-A may further enhance the clinical utility of sFlt1: PIGF ratio in predicting fetal growth restriction in patients with pre-eclampsia [43]. Other biomarkers associated with increased risk for pre-eclampsia later in pregnancy include elevated leptin levels, decreased serum adiponectin levels and elevated C-reactive protein levels. Furthermore, it cannot be excluded that the ratio adiponectin/leptin is possibly more important rather than the individual serum levels of the two adipokines in obesity and obese women. Similarly, it is not fully elucidated how leptin affects trophoblast implantation, and to which extent this effect contributes to pre-eclampsia development. The literature review also produced a consistent body of evidence about the roles of oxidative stress, peroxynitrite production, and decreased eNOS enzymatic activity in impaired placentation and pre-pre-eclampsia development [66], further supported by the potential evidence of a regulatory role by adiponectin on ROS formation [68,69]. Predictably, supplementation with NO donors or L-arginine, and inhibition of type-5 phosphodiesterase, which normally promotes cGMP degradation and limits NO-cGMP functionality, are actively pursued as therapeutic approaches to attenuate oxidative stress and vasoconstriction, and improve uterine vascularization [66]. Furthermore, a possible interplay between leptin and adiponectin on one hand, and ROS formation and alterations in other biomarkers for pre-eclampsia on the other hand, may exist, further emphasizing the possible role of dysmetabolism in adipocytes, and the onset and progression of pre-eclampsia in overweight pregnant women.

In contrast to the robust support from the literature on the predictive relevance of adiponectin and CRP levels for pre-eclampsia, recent literature reports inconsistent results for resistin and visfatin. Hence, for these markers of interest more research is still warranted. In considering the elevated C-reactive protein levels observed in pre-eclampsia cases as a result of systemic inflammation, it would be interesting to better investigate the mechanism(s) by which C-reactive protein levels improve following exercise regimens [45]. Although the improvement observed was not statistically significant [45], it is possible that exercise in the context of a holistic approach that includes diet and weight loss could potentially reduce systemic inflammation and consequently C-reactive protein levels and ameliorate the circulating levels of leptin and adipokines, improving pregnancy health in general.

Lastly, the importance of HLA-G levels in predicting pre-eclampsia merits more in-depth investigation. The histocompatibility antigen HLA-G appears to facilitate correct invasion of the cytotrophoblast [58], and its expression is reduced in all forms of pre-eclampsia, although a recent study [46] has not identified a strong relation between expression of this antigen and the incidence of pre-eclampsia, in particular in the decidual acute atherosis pre-eclampsia subtype. The small sample size of the study (83 women only) precludes extrapolation to the broader pre-eclampsia population, and warrant further investigation of HLA-G levels in a larger study population.

In this review, we have collated recent literature which provides evidence that confirms obesity to be a major risk factor for pre-eclampsia. The literature reviewed also suggests that the abnormalities in the levels of adiponectin, leptin, and possibly other cytokines as observed under the dysmetabolic conditions associated with weight gain and frank obesity may play an important role, albeit not fully understood, in the pathophysiological processes leading to pre-eclampsia. More detailed studies conducted in larger sample sizes are certainly necessary to understand the role adipokines play in the development of pre-eclampsia in obese pregnant women or in women who experience an excessive weight gain during their pregnancies.

## Figures and Tables

**Table 1:** The articles used for this review are ordered by the reference number by which the articles were introduced and discussed in the manuscript (see list of abbreviations at the end of the review for clarification).

Researchers (Reference)	Publication Date	Country	Sample	Study Type	Goal	Findings
Heslehurst, N.; Simpson, H.; Ells, L.J.; Rankin, J.; Wilkinson, J.; Lang, R.; Brown, T.J.; Summerbell, C.D. [9]	2008	UK	Obese women with proper comparison group	Meta-analysis of cohort studies of pregnant women from 16 weeks of pregnancy to delivery	To investigate relationships between obesity and impact on obstetric care	The study shows a significant relationship between obesity and increased odds of C-section and other delivery complications including longer duration of hospital stay and increased neonatal intensive care requirement. Maternal obesity significantly contributes to a poorer prognosis for mother and baby
Yao, R.; Ananth, C.V.; Park, B.Y.; Pereira, L.; Plante, L.A. [10]	2014	US	Almost 3 million singleton births were evaluated	Population-based retrospective cohort study	To examine the association of maternal pre-pregnancy body mass index and risk of stillbirth	The results of this study indicate an increase in the risk of stillbirth with increasing BMI. The association is strongest at early- and late-term gestation periods. Extreme maternal obesity is a significant risk factor for stillbirth
Afshin, A.; Forouzanfar, M.H.; Reitsma, M.B.; Sur, P.; Estep, K.; Lee, A.; Marczak, L.; Mokdad, A.H.; Moradi-Lakeh, M.; Naghavi, M. et al. [11]	2017	195 countries	Data from 68.5 million persons in 195 countries	Data analysis: 1990 to 2015	To assess the trends in the prevalence of overweight and obesity among children and adults between 1980 and 2015	The study shows that more than two thirds of deaths related to high BMI were due to cardiovascular disease
Lisonkova, S.; Joseph, K.S. [12]	2013	US	456,668	Cox and logistic regression models	To examine the gestational age–specific incidence of pre-eclampsia onset and identify the associated risk factors and birth outcomes	The study indicates that early-and late-onset pre-eclampsia shares some etiological features, differ with regard to several risk factors, and lead to different outcomes. The 2 pre-eclampsia types should be treated as distinct entities from an etiological and prognostic standpoint
Rasmussen, S.; Irgens, L.M.; Espinoza, J. [13]	2014	Norway	77,294 singleton pregnancies	Observational study comparing birthweight percentiles and z-scores	To assess whether excess of LGA neonates in pre-eclamptic women delivering at term is attributable to maternal obesity	The study indicates that accelerated fetal growth is observed in a subset of pre-eclamptic women delivering at term. The excess of LGA neonates is attributable to maternal obesity
Mbah, A.; Kornosky, J.; Kristensen, S.; August, E.; Alio, A.; Marty, P.; Belogolovkin, V.; Bruder, K.; Salihu, H. [14]	2010	US	All singleton live births in the state of Missouri from 1989 to 2005	Logistic regression models with adjustment for intra-cluster correlation	To determine the association between obesity and the risk of pre-eclampsia	The study shows that the rate of pre-eclampsia increases with increasing BMI. Obese women (BMI ≥ 30 kg/m^2^) have a higher risk for pre-eclampsia. Super-obese women have the highest incidence (13.4%)
Bodnar, L.M.; Catov, J.M.; Klebanoff, M.A.; Ness, R.B.; Roberts, J.M. [15]	2007	US	38,188 pregnant women	Prospective cohort study	To assess the association of pre-pregnancy BMI with severe and mild pre-eclampsia and transient hypertension of pregnancy	The study identifies a monotonic, dose-response relation between pre-pregnancy BMI and risk of both severe and mild pre-eclampsia as well as the risk of severe and mild transient hypertension of pregnancy
Bodnar, L.M.; Ness, R.B.; Markovic, N.; Roberts, J.M. [16]	2005	US	1179 primiparous women	Prospective cohort study	To explore the relation between pre-pregnancy BMI and the risk of pre-eclampsia	The study indicates that the risk of pre-eclampsia rises with increasing pre-pregnancy body mass index (from 15 to 30)
Abalos, E.; Cuesta, C.; Carroli, G.; Qureshi, Z.; Widmer, M.; Vogel, J.P.; Souza, J.P. [17]	2014	Multi-country: 29 countries from Africa, Asia, Latin America and the Middle East.	357 health facilities conducting 1000 or more deliveries annually	Secondary analysis of the WHOMCS database	To assess the incidence of hypertensive disorders of pregnancy and related severe complications	This WHOMCS on maternal and newborn health research network identifies hypertensive disorders of pregnancy as pre-eclampsia, eclampsia as adverse, life-threatening maternal and perinatal outcomes
Zhang, J.; Meikle, S.; Trumble, A. [19]	2003	US	300,000 deliveries assessed	Data Analysis	To study the incidence of severe maternal morbidity associated with hypertensive disorders of pregnancy in the US	The study shows that pre-eclampsia and eclampsia carry a high risk for severe maternal morbidity. Compared to Caucasians, African Americans have higher incidence of hypertensive disorders in pregnancy and suffer from more severe complications
Funai, E.F.; Friedlander, Y.; Paltiel, O.; Tiram, E.; Xue, X.; Deutsch, L.; Harlap, S. [20]	2005	Israel	37,061 women	Cox-proportional model	To investigate the long-term risk of mortality in women with pre-eclampsia	The study indicates that among women with pre-eclampsia who have subsequent births without pre-eclampsia, the excess risk of mortality became manifest only after 20 years
Wahabi, H.A.; Fayed, A.A.; Alzeidan, R.A.; Mandil, A.A. [21]	2014	Saudi Arabia	2701 women	Retrospective Study	To investigate the independent effect of GDM and obesity on the adverse pregnancy outcomes at term	The study shows a significant increase in the percentage of macrosomia, high birth weight, and pre-eclampsia in women with GDM and obesity. The study also shows a two- fold increase in C-section delivery in obese women
Mrema, D.; Lie, R.T.; Østbye, T.; Mahande, M.J.; Daltveit, A.K. [22]	2018	Tanzania	17,738 singleton birth women	Multi-variable analysis of registry based data	To examine the association between pre pregnancy BMI and the risk of pre-eclampsia in Tanzania	The study indicates that pre-pregnancy maternal overweight and obesity are associated with an increased risk of pre-eclampsia
Sohlberg, S.; Stephansson, O.; Cnattingius, S.; Wikström, A.K. [23]	2012	Sweden	503,179 nulliparous women	Population-based Cohort study	To determine whether BMI has an effect on pre-eclampsia of all severities	The study shows that short maternal stature and high BMI increase risks of pre-eclampsia of all severities. The associations is especially strong between short stature and severe types of pre-eclampsia, and high BMI and mild types of pre-eclampsia
Dantas, E.M.D.M.; Pereira, F.V.M.; Queiroz, J.W.; Dantas, D.L.D.M.; Monteiro, G.R.G.; Duggal, P.; Azevedo, M.D.F.; Jeronimo, S.M.B.; Araujo, A.C.P.F. [24]	2013	Brazil	242 women	Prospective case control study	To determine the frequency of and risk factors for pre-eclampsia in a low income population	The study indicates that women with pre-eclampsia develope chronic hypertension more often than normotensive controls
Shi, P.; Liu, A.; Yin, X. [25]	2021	China	1606 with GDM	Retrospective Cohort	To examine association between gestational weight gain in women with GDM and adverse pregnancy outcomes	The study indicates higher risk for pre-eclampsia and pregnancies complicated by hypertension in women with higher BMIs and high rates of gestational weight gain (above IOM guidelines)
Shao, Y.; Qiu, J.; Huang, H.; Mao, B.; Dai, W.; He, X.; Cui, H.; Lin, X.; Ly, L.; Wang, D.; Tang, Z.; Xu, S.; Zhao, N.; Zhou, M.; Xu, X.; Qiu, W.; Liu, Q.; Zhang, Y. [26]	2017	Lanzhou, China	9516	Cohort Study	To evaluate independent and joint effects of pre-pregnancy BMI and GWG on pre-eclampsia and its subtypes	The study shows that women overweight or obese have an increased risk for pre-eclampsia. Women with higher GWG also present with increased risk for pre-eclampsia. Similar increased risk was reported for all subtypes of pre-eclampsia. The highest risk for pre-eclampsia was observed to be directly proportional to the level of weight gain during gestation
Endershaw, M.; Abebe, F.; Worku, S.; Menber, L.; Assress, M.; Assefa, M. [27]	2016	Northwest Ethiopia	151 Pregnant women; 302 controls	Case-control study	To estimate the effect of obesity and dietary habits on pre-eclampsia	The study indicates that the risk of pre-eclampsia is higher among obese women compared to leaner women. The effect of obesity on pre-eclampsia is significant in women younger than 35 y.o. Folate supplementation is associated with a reduced risk of pre-eclampsia.
Meander, L.; Lindqvist, M.; Mogren, I.; Sandlund, J.; West, C.E.; Domellöf, M. [28]	2021	North Sweden	2203	Epidemiological study	To examine the level of physical activity and sedentary time in the Sweden population and explore effects of gestation weight gain, mode of delivery, birth weight of the child, and blood loss	The study shows that higher levels of physical activity are associated with reduced risk of emergency C-section and low gestational weight gain. Only 27.3% of the women considered in the sample achieve recommended level of physical activity, which is associated with more favorable pregnancy outcomes
Borgen, I.; Aamodt, G.; Harsem, H.; Haugen, M.; Meltzer, H.M.; Brantsaeter, A.L. [29]	2012	Norway	32,933 nulliparous women	Mother and Child Cohort Study	To determine whether maternal sugar consumption increases the risk of pre-eclampsia in nulliparous Norwegian women	The study indicates that sugar-sweetened carbonated and non-carbonated beverages are significantly associated with increased risk of pre-eclampsia, both independently and combined
Schoenaker, D.A.J.M.; Soedamah-Muthu, S.S.; Callaway, L.K.; Mishra, G.D. [30]	2015	Australia	292 GDM	population-based cohort study	To examine the associations between pre-pregnancy dietary patterns and the incidence of GDM	The study shows that the ‘Meats, snacks and sweets’ pattern is associated with higher GDM risk after adjustment for socioeconomic, reproductive and lifestyle factors
Clausen, T.; Slott, M.; Solvoll, K.; Drevon, C.A.; Vollset, S.E.; Henriksen, T. [31]	2001	Norway	3133 women	prospective, population-based, cohort study of pregnant women	To investigate prospectively whether diet in the first half of pregnancy is associated with risk for pre-eclampsia	The study indicates that high intake of energy, sucrose, and polyunsaturated fatty acids is associated with increased risk of pre-eclampsia
Kibret, K.T.; Chojenta, C.; Gresham, E.; Tegegne, T.K.; Loxton, D. [32]	2018	Australia	21 studies were assessed	A systematic review and meta-analysis	To assess the association between dietary patterns and the risk of adverse pregnancy and birth outcomes	The study shows that dietary patterns with a higher intake of fruits, vegetables, legumes, whole grains and fish are associated with a decreased likelihood of adverse pregnancy and birth outcomes
Schoenaker, D.A.J.M.; Soedamah-Muthu, S.S.; Mishra, G.D. [33]	2014	Australia	In total, 23 cohort and 15 case-control studies were identified	systematic review and meta-analyses	To determine whether dietary factors play a role in the prevention of HDP	The study indicates that higher total energy and lower magnesium and calcium intake measured during pregnancy are identified as related to HDP
Ikem, E.; Halldorsson, T.; Birgisdottir, B.; Rasmussen, M.; Olsen, S.; Maslova, E. [34]	2019	Denmark	55,139 Danish women	Prospective Longitudinal Study	To examine the association between mid-pregnancy dietary patterns and PAH	The study shows a protective association of seafood diet and a harmful association of Western diet with PAH
Wang, D.; Darling, A.M.; McDonald, C.R.; Perumal, N.; Liu, E.; Wang, M.; Aboud, S.; Urassa, W.; Conroy, A.L.; Hayford, K.T.; Liles, W.C.; Kain, K.C.; Fawzi, W.W. [35]	2021	Tanzania	1002 women	Prospective cohort study	To evaluate associations between a panel of inflammatory, angiogenic, and metabolic proteins measured in mid-pregnancy and gestational weight gain	The study shows that plasma concentrations of leptin at mid-pregnancy are associated with gestational weight gain among pregnant women in Tanzania
Molvarec, A.; Szarka, A.; Walentin, S.; Beko, G.; Karadi, I.; Prohazska, Z. Rigo, J., Jr [36]	2011	Hungary	Sixty pre-eclamptic patients, 60 healthy pregnant women and 59 healthy non-pregnant women	Case Control study	To investigate whether serum leptin levels are related to the clinical characteristics of healthy non-pregnant and pregnant women and pre-eclamptic patients	The study shows that serum leptin levels correlate inversely with fetal birth weight in healthy pregnant women. Elevated serum leptin concentrations directly correlate with adipose tissue mass, systemic inflammation, and systolic blood pressure, and negatively correlate with birth weight in normal pregnancies. In both normal and pre-eclampsia pregnancies, increased leptin levels correlate with interferon-y-inducible protein (IP-10) levels. Elevated serum leptin levels and sFlt-1/PlGF ratio have an additive effect on the risk of pre-eclampsia
Bawah, A.T.; Yeboah, F.A.; Nanga, S.; Alidu, H.; Ngala, R.A. [37]	2020	Ghana	90 PE	Case-control study	To determine the levels of serum adiponectin, leptin, resistin, visfatin, and lipids during the first trimester of pregnancy and to evaluate the relation between these markers and pre-eclampsia	The study shows the presence of significant differences in adipokines levels between the pre-eclampsia group and the group without pre-eclampsia. Adiponectin, leptin, resistin, and visfatin are identified as significant predictors of pre-eclampsia, with resistin being the best predictor after controlling for BMI
Hu, W.; Wang, Z.; Wang, H.; Huang, H.; Dong, M. [38]	2008	China	27 women with pre-eclampsia, 28 women in the third trimester of normal pregnancy, and 28 normal non-pregnant women	Case Control study	To characterize the changes in serum visfatin levels in late normal pregnancy and pre-eclampsia	The study indicates a decrease in visfatin level in pre-eclampsia, suggesting that visfatin and adipokine-associated metabolic abnormalities are involved in the pathogenesis of the disease
Kapustin, R.V.; Tcybuk, E.M.; Chepanov, S.V.; Alekseenkova, E.N.; Kopteeva, E.V.; Arzhanova, O.N. [39]	2021	Russia	140 pregnant women	Case Control study	To evaluate sFlt-1 and PlGF levels in the blood of pregnant women	The study shows that blood level alterations of PlGF and sFlt-1 are characteristic of patients with diabetes mellitus in the first and third trimesters of pregnancy. Determination of the sFlt-1/PlGF ratio is a valid method for predicting the development or absence of pre-eclampsia in women with diabetes mellitus
Nikuei, P.; Rajaei, M.; Roozbeh, N.; Mohsenu, F.; Poordarvishi, F.; Azas, M.; Haidari, S. [40]	2020	Iran	23 mild, 15 severe pre-eclamptic patients, and 20 normal term pregnant women	ROC curve analysis	To evaluate the diagnostic accuracy of sFlt-1 to PlGF ratio for diagnosis of pre-eclampsia in an Iranian population	The study shows that sFlt-1/PlGF ratio has higher accuracy than each individual parameter in differentiating pre-eclampsia patients from non-pre-eclampsia patients
Andraweera, P.; Dekker, G.; Roberts, C. [41]	2012	Australia	18 women with pre-eclampsia; 15 women with gestational hypertension; 13 normo-tensive women with SGA; 10 women with spontaneous pre-term birth, and 30 women with uncomplicated pregnancy	Retrospective analysis	To elucidate the role of angiogenic factors in placentation and to evaluate the predictive value of their protein concentrations and genetic variations in pregnancy complications	The study concludes that the current predictive value of the VEGF family as biomarkers appears to be limited to early-onset pre-eclampsia
León-Reyes, G.; Maida-Claros, R.F.; Urrutia-Medina, A.X.; Jorge-Galarza, E.; Guzman-Grenfell, A.M.; Fuentes-Garcia, S.; Medina-Navarro, R.; Moreno-Eutimio, M.A.; Muñoz-Sánchez, J.L.; Hicks, J.J.; Torres-Ramos, Y.D. [42]	2017	Mexico	Thirty women diagnosed with pre-eclampsia and thirty women without pre-eclampsia were included in the study	Transversal and Observational	To evaluate the oxidative profile of lipoproteins isolated from women with pre-eclampsia	The study demonstrates evident oxidative changes in the lipids and proteins in HDL-c and LDL-c particles in PE women
Sharabi-Nov, A.; Srsen, T.P.; Kumer, K.; Vodusek, V.F.; Fabjan, T.; Tul, N.; Meiri, H.; Nicolaides, K.H.; Osredkar, J. [43]	2021	Slovenia	31 cases of pre-eclampsia, 16 of FGR, 42 of pre-eclampsia + FGR, 15 preterm delivery, and 21 unaffected controls	Secondary Analysis	To examine the potential additive value of maternal serum Inhibin-A	The study shows that maternal serum Inhibin-A augments the value of maternal serum PIGF and sFlt-1/PIGF ratio to predict pre-eclampsia near delivery
Kumer, K.; Sharabi-Nov, A.; Vodusek, V.F.; Srsen, T.P.; Tul, N.; Fabian, T.; Meiri, H.; Nicolaides, K.H.; Osredkar, J. [44]	2021	Slovenia	31 cases of pre-eclampsia, 16 of FGR, 42 of pre-eclampsia + FGR, 15 cases who developed with unrelated complications before 37 weeks, and 21 unaffected controls	Secondary Analysis	To assess the accuracy of PlGF, sFlt-1, and sEng in the diagnosis of suspected pre-eclampsia with and without FGR near delivery	The study shows that pro- and anti-angiogenic markers are important clinical tools to identify pre-eclampsia near delivery even in the absence of changes in FGR
Hawkins, M.; Braun, B.; Marcus, B.H.; Stanek, E.; Markenson, G.; Chasan-Taber, L. [45]	2015	US	171 women divided into 84 in exercise protocol and 87 wellness protocol	Randomized control trial	To evaluate the impact of an individually-tailored motivationally-matched exercise intervention on CRP in pregnant women	The study shows that CRP decreases from pre-to post-intervention in the exercise arm and increases in the health and wellness arm; however, the between group difference is not statistically significant (*p* = 0.14). Findings do not differ according to ethnic group or pre-pregnancy body mass index
Johnsen, G.M.; Fjeldstad, H.E.S.; Drabbels, J.J.M.; Haasnoot, G.W.; Eikmans, M.; Størvold, G.L.; Alnaes-Katjavivi, P.; Jacobsen, D.P.; Scherjon, S.A.; Redman, C.W.G.; Claas, F.H.J.; Staff, A.C. [46]	2021	Norway	83 normo-tensive and 83 pre-eclamptic pregnancies	Case Control study	To investigate whether variants of the 3′UTR of the HLA-G gene in mother and fetus are associated with acute atherosis, a pregnancy specific arterial lesion of the decidua basalis that is prevalent in pre-eclampsia	The study shows that HLA-G polymorphisms in the fetus are associated with acute atherosis. These polymorphisms lead to altered HLA-G expression in the decidua basalis, affecting local feto-maternal immune tolerance and development of acute atherosis in pre-eclampsia

## Data Availability

Not applicable.

## Abbreviations

Terminology and Abbreviations (in alphabetical order)	
BMI	Body mass index
CDC	Center for disease control
CRP	C-reactive protein
sENG	Serum Endoglin
FGR	Fetal growth restriction
sFlt-1	Circulating soluble fms-like tyrosine kinase-1
GDM	Gestational diabetes mellitus
GWG	Gestational weight gain
HDL	High Density Lipoprotein
HDP	Hypertensive disorders of pregnancy
HLA-G	Human leukocyte antigen Class I, G
HTN	Hypertension
IOM	International Organization for Migration
LDL	Low Density Lipoprotein
LGA	Large for gestational age
PAH	Pregnancy associated hypertension
PE	Pre-eclampsia
PIGF	Placental growth factor
SGA	Small for gestational age
VEGF	Vascular endothelial growth factor
WHO	World health organization
WHOMCS	WHO multi-country survey
